# Viability-Resolved Metagenomics Reveals Antagonistic Colonization Dynamics of Staphylococcus epidermidis Strains on Preterm Infant Skin

**DOI:** 10.1128/mSphere.00538-21

**Published:** 2021-09-15

**Authors:** K. Taylor Hellmann, Carly E. Tuura, James Fish, Jaimin M. Patel, D. Ashley Robinson

**Affiliations:** a Department of Pathology, University of Mississippi Medical Center, Jackson, Mississippi, USA; b Department of Pediatrics, University of Mississippi Medical Center, Jackson, Mississippi, USA; c Department of Microbiology and Immunology, University of Mississippi Medical Center, Jackson, Mississippi, USA; University of Nebraska Medical Center

**Keywords:** metagenomics, preterm infants, *Staphylococcus epidermidis*, coagulase-negative staphylococci

## Abstract

Preterm infants are at increased risk of infections caused by coagulase-negative staphylococci (CoNS) that colonize skin. Technical barriers in sequencing low-microbial-biomass skin swabs from preterm infants hinder attempts to gain a strain-level understanding of CoNS colonization dynamics within their developing skin microbiome. Here, the microbiome of five skin sites and available stool was studied from four preterm infants hospitalized over their first 2 months of life. We used propidium monoazide treatment of samples to enrich for the viable microbiome and metagenomic shotgun sequencing to resolve species and strains. The microbiome of different skin sites overlapped with each other, was dominated by the CoNS species Staphylococcus epidermidis and Staphylococcus capitis, and was distinct from stool. Species diversity on skin increased over time despite antibiotic exposure. Evidence of antagonism between the most common S. epidermidis strains, ST2 and ST59, included negative relationships for species correlation networks and *in situ* replication rates and that ST2 colonized skin earlier but was often replaced by ST59 over time. Experiments done with reference isolates showed that ST2 produced more biofilm than ST59 on plastic surfaces, which was reduced in mixed culture. We also discovered that a rare S. epidermidis strain, ST5, grew rapidly in stool in association with Stenotrophomonas maltophilia from a suspected episode of infection. Viability treatment of samples and moderate throughput shotgun sequencing provides strain-level information about CoNS colonization dynamics of preterm infant skin that ultimately might be exploited to prevent infections.

**IMPORTANCE** The skin is a habitat for microbes that commonly infect preterm infants, but the use of sequencing for fine-scale study of the microbial communities of skin that develop in these infants has been limited by technical barriers. We treated skin swabs of preterm infants with a photoreactive dye that eliminates DNA from nonviable microbes and then sequenced the remaining DNA. We found that two strains of the most common species, Staphylococcus epidermidis, showed an antagonistic relationship on skin by cooccurring with different species, replicating fastest in different samples, and dominating skin sites at different times. Representatives of these strains also differed in their ability to stick to plastic surfaces—an important pathogenicity trait of this species. Our study shows the feasibility of gaining detailed information about strain colonization dynamics from this difficult-to-sequence body site of preterm infants, which might be used to guide novel approaches to prevent infections.

## INTRODUCTION

Among the health problems associated with preterm birth is an increased risk of infections. Bloodstream infections that occur >72 h postbirth are more likely for preterm infants of very low birthweight (<1,500 g) or gestational age (<32 weeks) and bring an increased risk of neurodevelopmental sequelae and death (1, 2). Such infections are facilitated by the impaired innate immunity of preterm infants and the bloodstream access provided by intravascular catheters that are used during neonatal intensive care. *Staphylococcus* species, especially coagulase-negative staphylococci (CoNS), such as Staphylococcus epidermidis and Staphylococcus capitis, account for 45 to 70% of these infections (3–5). Although the CoNS are part of the microbial community of the skin of healthy infants and adults, they are a major cause of antimicrobial-resistant bloodstream infections in preterm infants (3, 4, 6, 7).

One strategy to address the broader problem of antimicrobial-resistant bacterial infections in susceptible patient populations is to learn how to modulate the composition of microbial communities to promote the growth of commensals that are antagonistic to pathogens (8–10). Some S. epidermidis strains may be useful as probiotics through their production of antimicrobial compounds that inhibit more aggressive pathogens, such as Staphylococcus aureus, that also live on skin and adjacent surfaces (9, 11). Other S. epidermidis strains may encode pathogenicity traits and be more likely to cause infections (12–14). Thus, a probiotic strategy to curtail CoNS infections in preterm infants would depend on a strain-level understanding of their developing skin microbiome, including strain-specific antagonistic and mutualistic interactions.

The taxonomic resolution provided by the frequently sequenced V3 to V5 regions of 16S rRNA is inadequate to identify CoNS species (15), let alone strains, within the microbiome. Metagenomic shotgun sequencing can identify CoNS species and strains (16, 17), but this approach has been focused on the characterization of the preterm infant gut microbiome (18–20). Technical barriers must be overcome in order to use sequencing to obtain strain-level information about the preterm infant skin microbiome. This body site is generally of low microbial biomass for which sequencing of skin swabs may be expected to yield >90% human and <10% microbial sequences (21, 22). This sampling limitation is compounded by the fragility of preterm infant skin that precludes the use of aggressive swabbing techniques and chemicals that might otherwise produce a higher yield of both human and bacterial sequences (22). Thus, it is not clear how much sequencing would be required to identify CoNS strains from preterm infant skin swabs.

Frequent medical interventions, such as the use of systemic antibiotics along with antiseptic wipes and baths, may result in bacterial DNA on preterm infant skin that derives from dead and dying bacterial cells as well as live bacterial cells. This issue of the viability of the bacteria at the time of microbiome sampling is rarely addressed, but the presence of DNA from nonviable bacteria may confound attempts to understand interactions between cocolonizing strains. One approach to focus on the viable microbiome is to treat samples with a dye such as propidium monoazide (PMA), which intercalates into DNA that is accessible extracellularly and in nonviable cells with compromised membranes (23). In a light-sensitive reaction, the PMA will covalently cross-link to the DNA and, thus, inhibit PCR amplification and sequencing. PMA has been used in earlier quantitative PCR studies (24–26) and more recently for sequencing the viable microbiome from cleanrooms and saliva samples (21, 27).

The purpose of this study was to focus microbiome sampling on multiple different skin sites from four longitudinally followed preterm infants over their first 2 months of life in order to better understand CoNS colonization dynamics. PMA treatment of samples was used to enrich for the viable microbiome. Moderate throughput shotgun sequencing provided adequate resolution to identify two predominant S. epidermidis strains and to discern an antagonistic relationship between them.

## RESULTS

### Effects of propidium monoazide treatment on cultures of S. epidermidis.

**FIG 1:**
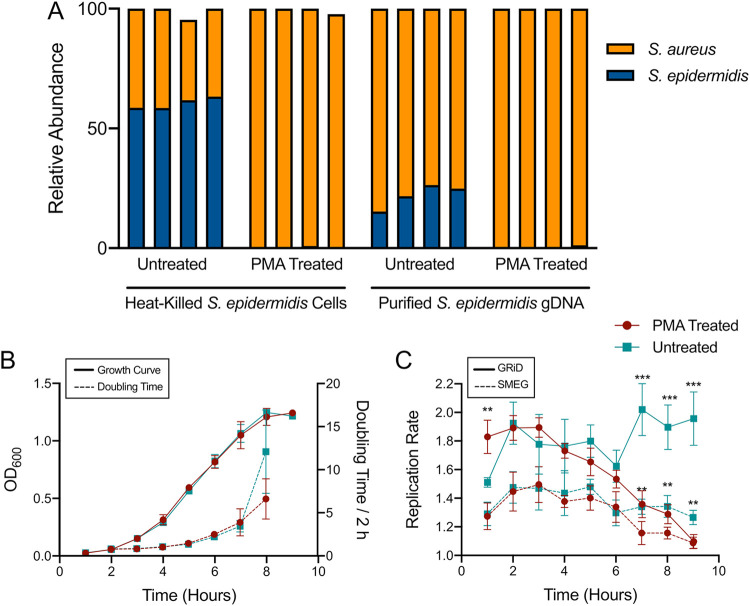
Effectiveness of PMA treatment on removing DNA from nonviable S. epidermidis. (A) Live S. aureus (orange) was combined with either heat-killed S. epidermidis cells (blue) or purified S. epidermidis gDNA (blue) prior to treatment in four replicates. (B) S. epidermidis growth measured by optical density (solid line) and doubling time (dashed line) in TSB. (C) S. epidermidis replication rate estimated by GRiD (solid line) or SMEG (dashed line) during the growth depicted in panel B. Each point in panels B and C represents the mean of four samples, and the error bars represent the standard deviation. The samples were either PMA treated (red circles) or left untreated (teal squares). *t* tests were used to compare mean replication rates by hour (**, *P* < 0.01; ***, *P* < 0.001).

Propidium monoazide (PMA) treatment may not remove DNA from nonviable bacteria equally well for all species (24–26). Thus, we sought to verify that PMA treatment could remove DNA from relatively high concentrations of nonviable S. epidermidis under laboratory conditions. We combined ∼5 × 10^8^ CFU/ml live S. aureus from broth culture with either an equal portion of heat-killed S. epidermidis or ∼250 ng purified genomic DNA from S. epidermidis. Cultures were treated with PMA, and control cultures were left untreated. Sequences from these samples were analyzed with MetaPhlAn2 (28) to determine the relative abundance of S. aureus and S. epidermidis. Compared to untreated controls, the PMA treatment removed 99.7% of the heat-killed S. epidermidis DNA and 98.6% of the purified genomic DNA from S. epidermidis (Fig. 1A).

We also examined whether PMA treatment affected estimates of replication rate from sequencing data. S. epidermidis was sampled hourly over 9 h of growth in broth cultures that were monitored by optical density (Fig. 1B). Cultures were treated with PMA and control cultures were left untreated. Sequences from these samples were analyzed with GRiD (29) and SMEG (30) to estimate the replication rate at the species level and strain level, respectively. These tools make use of the relationship between growth rate and the sequence coverage ratio between the chromosomal origin and terminus; rapidly growing cells have a higher origin/terminus coverage ratio than slowly growing cells (31). The untreated samples of S. epidermidis gave significantly higher replication rate estimates than the PMA-treated samples at hours 7 to 9 (*t* test at these hours are as follows: GRiD, *P* < 0.0006; SMEG, *P* < 0.006) (Fig. 1C). Consequently, the S. epidermidis replication rate estimates from the untreated samples did not correlate with doubling times (fit of 2nd order polynomial regression: GRiD, *R*^2^ = 0.09 and *P* = 0.52; SMEG, *R*^2^ = 0.428 and *P* = 0.22), whereas the replication rate estimates from the PMA-treated samples did correlate with doubling times (fit of 2nd order polynomial regression: GRiD, *R*^2^ = 0.959 and *P* = 0.0037; SMEG, *R*^2^ = 0.845 and *P* = 0.0048) (see Fig. S1 in the supplemental material).

10.1128/mSphere.00538-21.4 FIG S1 The relationship between replication rate and doubling time in S. epidermidis. These panels represent 2nd order polynomial regressions of the replication rate data from Fig. 1C and the doubling times from Fig. 1B. GRiD values are represented in panels A and C. SMEG values are represented in panels B and D. The samples treated with PMA are represented in red (bottom) and the samples left untreated are represented in teal (top). *R*^2^ and *P* values are given for the regressions. Download FIG S1, PDF file, 0.2 MB. Copyright © 2021 Hellmann et al. 2021 Hellmann et al. https://creativecommons.org/licenses/by/4.0/ This content is distributed under the terms of the Creative Commons Attribution 4.0 International license.

### Microbiome sampling characteristics of different skin sites of preterm infants.

Four preterm infants of extremely low birthweight (average 600 g) and gestational age (average 25.1 weeks) were enrolled during their first week of life by maternal consent for longitudinal sampling of their microbiome. During this study, these infants were receiving medical care in the level IV neonatal intensive care unit (NICU) at the University of Mississippi Medical Center. Clinical characteristics of these infants are listed in Table S1 in the supplemental material. Microbiome sampling occurred every other week from birth week to 2 months of age. Six body sites were sampled, including swabs from the axillary vault, inguinal crease, nares, periumbilical region, and upper chest, and a sample of stool was collected if available in a diaper within the day of the skin swabbing. The specimens were immediately processed according to the PMA treatment protocol to enrich for the viable microbiome, followed by DNA extraction and concentration, and metagenomic shotgun sequencing.

10.1128/mSphere.00538-21.1 TABLE S1 Clinical characteristics of preterm infants enrolled in this study. Download Table S1, XLSX file, 0.01 MB. Copyright © 2021 Hellmann et al. 2021 Hellmann et al. https://creativecommons.org/licenses/by/4.0/ This content is distributed under the terms of the Creative Commons Attribution 4.0 International license.

All 80 proposed skin swabs (four infants, four time points, five skin sites) were collected as well as 12 available stool samples for a total of 92 microbiome samples. Of these, 32 samples were excluded because they were either contaminated (*n* = 1), had a library concentration of <0.09 ng/μl (*n* = 27), or were sequenced but produced no microbial reads (*n* = 4). A total of 60 samples, including skin swabs (*n* = 48) and stool samples (*n* = 12), produced microbial reads and were further analyzed (Table 1). On average, 21.1 million (M) reads were collected from skin swabs of which 5.9 M or 38.2% were microbial. By comparison, an average of 22.8 M reads were collected from stool samples of which 22.6 M or 99.2% were microbial. Among the skin sites, the nares had the highest average DNA concentration and most often gave microbial sequences (14/16 swabs were positive). The inguinal crease and axillary vault had the lowest average DNA concentration but also frequently gave microbial sequences (12/16 and 9/16 swabs were positive, respectively) (Table 1). On average, the inguinal crease and axillary vault had the highest number and percentage of microbial reads among skin sites. In contrast, the periumbilical region gave microbial sequences least often (5/16 swabs were positive) and had the lowest average number of microbial reads (0.99 M) (Table 1).

**TABLE 1:** Microbiome sampling characteristics of different body sites

Body site or sample type	Avg (SD) DNA concn (ng/μl)^*a*^	Avg (SD) library concn (ng/μl)	Avg (SD) no. of reads (M)^*b*^	No. of microbial-positive samples^*b*^	Avg (SD) no. of microbial reads (M)^*c*^	Avg (SD) percentage of microbial reads^*c*^	Avg (SD) microbial read length (bp)^*c*^
Axillary vault	<0.05 (0.04)	1.48 (2.74)	12.31 (14.58)	9	7.87 (13.39)	62.91 (34.01)	116.84 (3.57)
Inguinal crease	<0.05 (0.03)	0.64 (0.92)	12.71 (18.94)	12	9.17 (10.94)	65.58 (27.50)	119.25 (3.49)
Nares	2.54 (4.14)	2.79 (4.89)	37.75 (27.05)	14	5.45 (9.78)	15.74 (27.98)	119.03 (5.83)
Periumbilical	0.27 (0.34)	0.50 (0.42)	10.66 (11.75)	5	0.99 (0.76)	17.75 (22.75)	120.47 (5.41)
Upper chest	<0.10 (0.13)	0.4 (0.35)	14.71 (13.84)	8	2.89 (3.77)	21.65 (23.19)	119.42 (6.97)
Skin combined	<0.80 (2.45)	1.37 (3.02)	21.06 (21.99)	48	5.93 (9.74)	38.24 (35.56)	118.89 (5.03)
Stool	7.91 (8.15)	19.57 (11.41)	22.76 (9.19)	12	22.56 (9.05)	99.23 (0.61)	123.18 (5.90)

a Measured after concentration with sodium acetate-ethanol plus glycogen. Detection limit was 0.025 ng/μl.

b Number of reads generated from Illumina NextSeq run.

c After filtering reads with KneadData.

### Skin microbiome diversity and comparison to stool.

**FIG 2:**
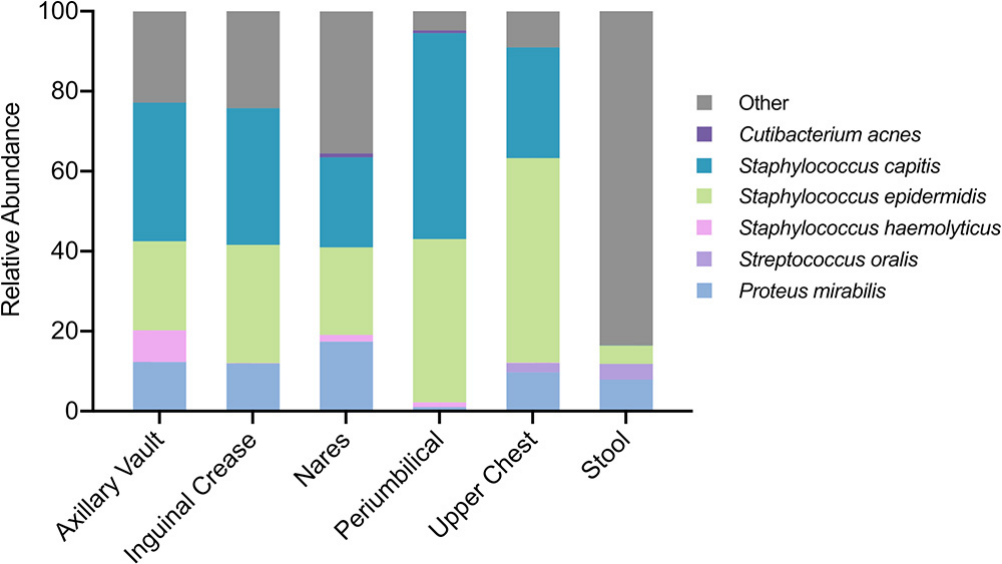
Average relative abundance of the six species present at all sites of preterm infant skin. Relative abundances of <1% may not be visible but are given in Table S2 in the supplemental material. Species not occurring at all skin sites were included in the “Other” category.

Based on MetaPhlAn2 analysis, a total of 87 bacterial and fungal species were detected among the 60 microbiome samples. Fifty-seven species occurred in more than one sample. Six species (Cutibacterium acnes, Staphylococcus capitis, Staphylococcus epidermidis, Staphylococcus haemolyticus, Streptococcus oralis, Proteus mirabilis) occurred at all five skin sites (Fig. 2; complete relative abundance given in Table S2 in the supplemental material). By a large margin, S. epidermidis and *S. capitis* occurred in the most skin swabs (47/48 and 37/48, respectively) and at the highest average relative abundance (30.7% and 31.6%, respectively). Furthermore, S. epidermidis was detected in the most stool samples (8/12) but with lower average relative abundance (4.5%) than Enterococcus faecalis (7/12 samples; 13% average relative abundance) and Klebsiella pneumoniae (6/12 samples; 7.9% average relative abundance).

10.1128/mSphere.00538-21.2 TABLE S2 Relative abundance of species identified by MetaPhlAn2. Download Table S2, XLSX file, 0.03 MB. Copyright © 2021 Hellmann et al. 2021 Hellmann et al. https://creativecommons.org/licenses/by/4.0/ This content is distributed under the terms of the Creative Commons Attribution 4.0 International license.

**FIG 3:**
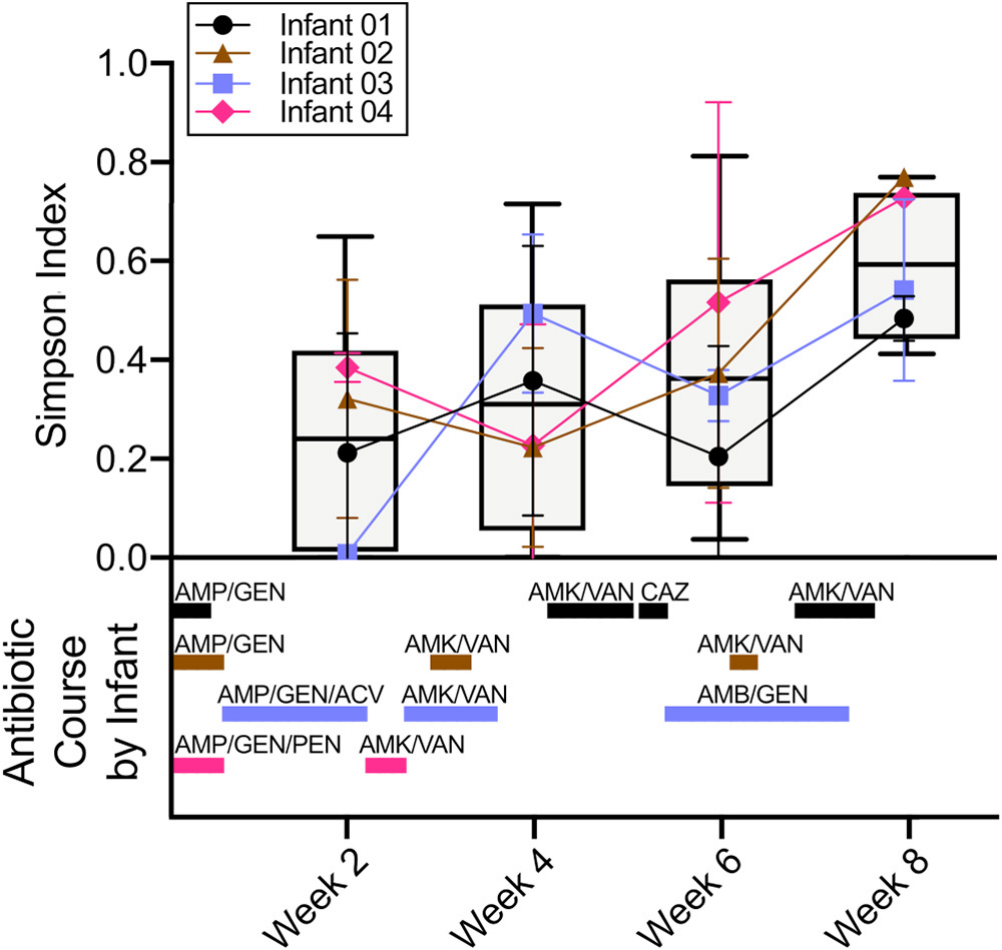
Microbiome diversity on preterm infant skin over time. (Top) Average Simpson index of species in skin swabs over time and for each infant shown with a line graph. Each infant is represented by a different color and symbol, and each point represents the average of the Simpson index from that week. The error bars represent the standard deviation. Boxplots represent the Simpson index for all infants combined. (Bottom) The duration of antibiotic treatments given to the infants during the course of this study. Each infant is represented by the same colors as the top panel. AMP, ampicillin; GEN, gentamicin; AMK, amikacin; VAN, vancomycin; CAZ, ceftazidime; ACV, acyclovir; AMB, amphotericin B; PEN, penicillin.

No significant difference in alpha or beta diversity was detected among the five skin sites, respectively; this finding justified the pooling of skin sites in subsequent analyses. Beta diversity as measured by the Jaccard index between skin and stool was significantly different (permutational multivariate analysis of variance [PERMANOVA], *P* < 0.001). In addition, a significant difference in alpha diversity as measured by the Simpson index was detected over time on the skin (analysis of variance [ANOVA], *P* = 0.027). The Simpson index increased an average of 2.5× between the first and last time points (test for linear trend, *P* = 0.0052), which spanned a period of antibiotic use in all four infants (Fig. 3). There was also a significant difference in beta diversity over time on the skin, with the Jaccard index indicating a different composition of species (PERMANOVA, *P* < 0.027). For example, K. pneumoniae was present more often in the last two time points.

### Strain profile for the most prevalent species, S. epidermidis.

Metagenomic assembly was done as an initial approach for S. epidermidis strain typing. However, only nine samples generated S. epidermidis genome bins, and only four of those were of acceptable quality (>70% completion and <5% contamination) (see Table S3 in the supplemental material). Among those four genomes, two strains were identified. One strain was multilocus sequence type 2 (ST2), accessory gene regulator (*agr*) type I, and positive for the intercellular adhesion (*ica*) biofilm-related genes, whereas the other strain was ST59, *agr* type I, and negative for *ica* genes. The ST2 and ST59 strains are genetically distinct, differing at 4/7 multilocus sequence typing loci and at >7,000 single nucleotide polymorphisms (SNPs) in the core genome, and they belong to different genetic clusters (12, 32). Interestingly, the amino acid sequences of the *agr* genes of these two strains were identical and presumably functional since no premature stop codons were detected. Antibiotic resistance gene content was variable, with *dfrC*, *fosB*, and *norA* identified in both strains and *mecA* and *mupA* identified in one genome of an ST2 strain (Table S3).

10.1128/mSphere.00538-21.3 TABLE S3 Metagenomic assembly and downstream analysis. Download Table S3, XLSX file, 0.02 MB. Copyright © 2021 Hellmann et al. 2021 Hellmann et al. https://creativecommons.org/licenses/by/4.0/ This content is distributed under the terms of the Creative Commons Attribution 4.0 International license.

**FIG 4:**
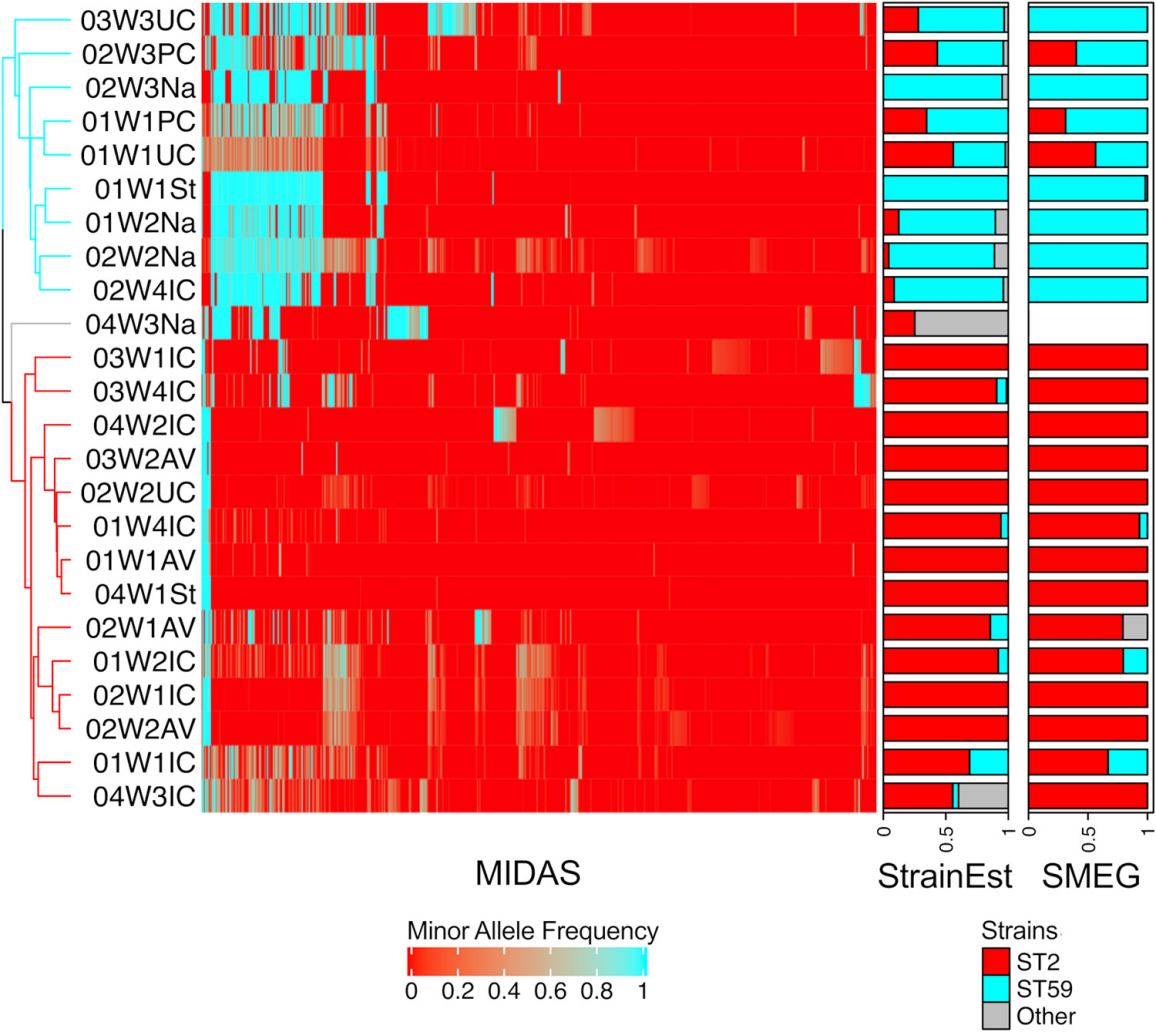
Identification of S. epidermidis strains in the preterm infant microbiome by three different methods. The heatmap was generated using the allele frequencies of 3,051 biallelic SNPs identified in the samples by MIDAS after quality filtering with default parameter values. The dendrogram on the left represents complete linkage clustering of allele frequencies based on Euclidean distances and is colored according to strain. On the right are bar charts representing the proportions of each strain identified in the samples by StrainEst and SMEG, respectively. ST2 is represented in red, ST59 is represented in cyan, and other STs are represented in gray.

Three more sensitive methods of analysis (MIDAS, StrainEst, and SMEG) were also used to identify S. epidermidis strains. MIDAS is capable of identifying a predominant strain in a sample (33). MIDAS was applied to 24 samples with >3.5× S. epidermidis genome coverage as estimated by MetaPhlAn2, and it identified ST2 and ST59 as the predominant strains as well a minor strain based on allele frequencies at 3,051 biallelic SNPs (Fig. 4). StrainEst is capable of distinguishing among a mixture of strains and estimating their relative abundance (16). StrainEst was applied to 33 samples with >1× S. epidermidis coverage as estimated by MetaPhlAn2. StrainEst identified ST2 and ST59 as the predominant strains and ST5 and ST218 as minor strains (Fig. 4). SMEG is also capable of distinguishing among a mixture of strains and can estimate their relative coverages and replication rates (30). SMEG was applied to the same 33 samples as StrainEst and identified two predominant phylogenetic clusters and one minor cluster; the predominant clusters contained ST2 and ST59, and the minor cluster contained ST5 (Fig. 4). The concordance among these three strain-typing methods in identifying the predominant strain was almost perfect, except for one upper chest skin swab where both StrainEst and SMEG identified a slightly higher proportion of ST2 than ST59 but MIDAS identified the predominant strain as ST59 (sample 01W1UC in Fig. 4).

### An antagonistic relationship between S. epidermidis strains on skin.

Ecological interactions between species can be inferred based on correlations in relative abundance data. To avoid spurious correlations, we used the sparse correlations for compositional data (SparCC) method to address the statistical issues of using relative abundance data for this analysis (34), and we focused on the skin samples to address the preferences of some species for certain body sites (35). We decomposed S. epidermidis relative abundance into the strain-specific relative abundance as estimated by StrainEst and then made comparisons with the other species. A statistically significant negative correlation occurred between ST2 and ST59 (*r* = −0.437; *P* = 0.035). Moreover, a positive, trending correlation occurred between ST2 and *C. acnes* (*r* = 0.447; *P* = 0.07) and between ST59 and S. oralis (*r* = 0.341; *P* = 0.06). The *in situ* replication rates of ST2 and ST59 in skin swabs were also negatively correlated with each other, although this was not statistically significant (7 samples with replication rate for both strains, *r* = −0.490 and *P* = 0.26; extended analysis to 32 samples where values were set to 1 for strains that had no detectable growth, *r* = −0.265 and *P* = 0.19).

**FIG 5:**
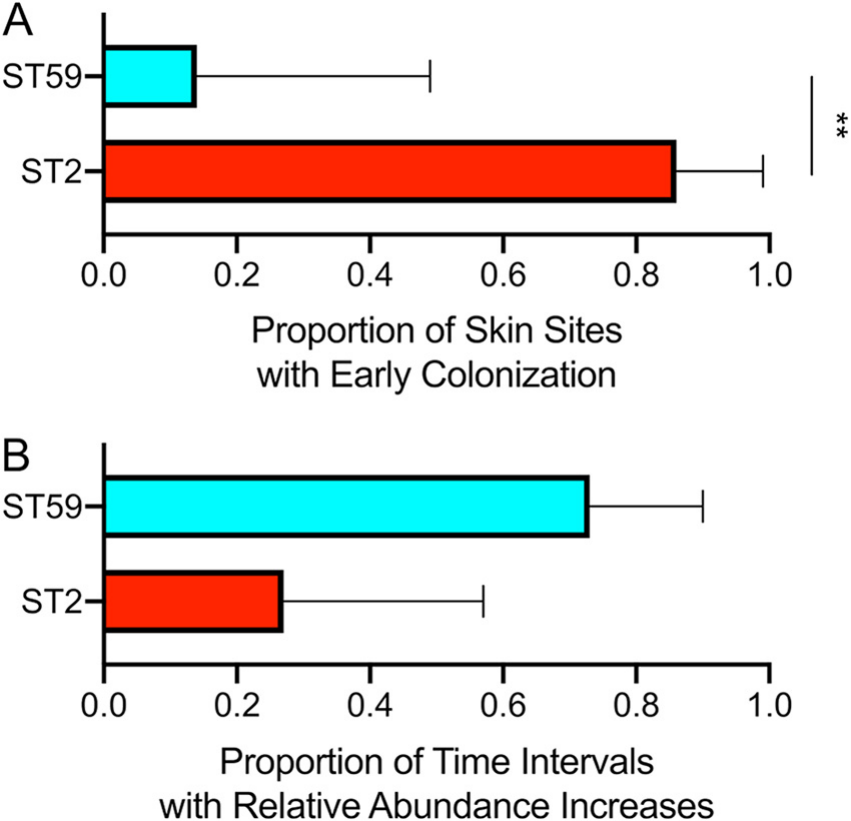
Colonization dynamics of two predominant strains of S. epidermidis on preterm infant skin. (A) Skin sites predominantly colonized early by a strain as determined by StrainEst. To be considered predominant, the strain had to have >50% relative abundance and be >10% more abundant than the other strains at the first time point they appeared. (B) Time intervals where the relative abundance of a strain increased as determined by StrainEst. Bars represent 95% confidence intervals. Test of proportions (**, *P* < 0.01).

We exploited the longitudinal study design to further examine the skin colonization dynamics of the two predominant S. epidermidis strains on preterm infant skin. First, the timing of skin colonization was examined. To establish early colonization, we considered that the strain must have a relative abundance of >50% plus be >10% more abundant than the other strains in the earliest sample obtained for a given infant and skin site. In this analysis, ST2 showed a significantly greater proportion of skin samples with early colonization compared to ST59 (test of proportions, *P* < 0.01) (Fig. 5A). Next, the replacement of strains over time was examined. For adjacent time points (connecting time points with missing intervening samples), we noted whether a strain increased or decreased in relative abundance. In this analysis, ST59 increased relative abundance in 73% of time intervals compared to ST2, which increased relative abundance in 27% of time intervals (Fig. 5B), though this was not statistically significant.

**FIG 6:**
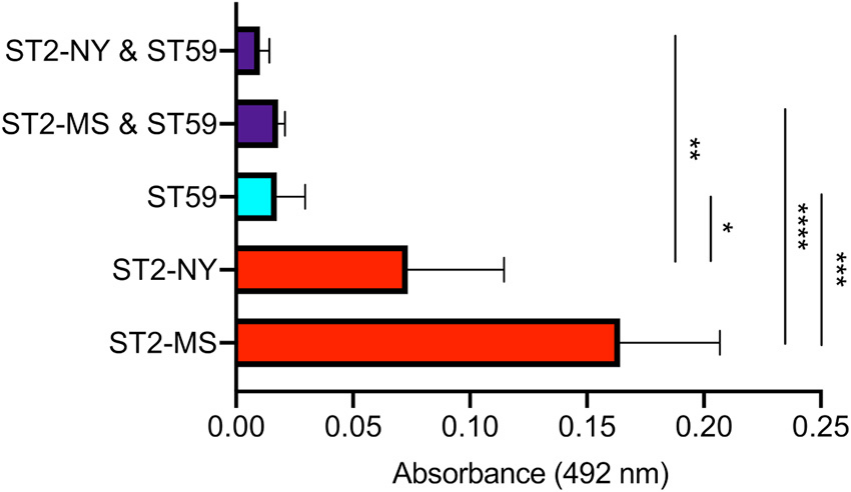
Ability of S. epidermidis strains to form biofilm in pure and mixed cultures. ST2 and ST59 strains were grown either separately in pure cultures (ST2 in red, ST59 in cyan) or in a mixture (purple). Crystal violet staining of 24-h biofilm was measured at 492 nm. Each bar represents the mean and standard deviations of four replicates. *t* tests were used to compare mean biofilm in the pure and mixed cultures (*, *P* < 0.05; **, *P* < 0.01; ***, *P* < 0.001; ****, *P* < 0.0001).

To further study the antagonism between ST2 and ST59, we examined the ability of reference isolates of these strain backgrounds to produce biofilm on polystyrene plates in pure and mixed cultures. Since no cultured isolates were available from the infants, we studied two isolates of ST2 from different U.S. states and one isolate of ST59. The ST2 isolates both produced more biofilm than the ST59 isolate when grown in isolation (*t* test for ST2-NY versus ST59, *P* = 0.022; for ST2-MS versus ST59, *P* = 0.0008) (Fig. 6). For the ST2 isolates, the biofilm produced in mixed culture was 86 to 89% less than that produced in pure culture (*t* test for ST2-NY mixed versus pure culture, *P* = 0.0025; for ST2-MS mixed versus pure culture, *P* < 0.0001), but this was not the case for the ST59 isolate (*t* test for ST59 mixed with ST2-NY versus pure culture, *P* = 0.37; for ST59 mixed with ST2-MS versus pure culture, *P* = 0.59) (Fig. 6). Thus, the ST59 isolate reduced the otherwise higher biofilm produced by ST2 isolates. Similar CFU per milliliter values of ST2 and ST59 were recovered from the mixed biofilms (see Fig. S2 in the supplemental material) that were seeded with cultures of similar optical density, which suggests that the biofilm reduction was not a result of ST59 killing ST2.

10.1128/mSphere.00538-21.5 FIG S2 CFU recovered from biofilms of pure and mixed cultures. ST59 was mixed with either ST2-MS (A) or ST2-NY (B), and the 24-h biofilm was plated to measure CFU. Mixed ST59 is the CFU of ST59 from the mixed cultures, measured from the colonies on the erythromycin TSA plates. Mixed ST2-MS and mixed ST2-NY are the CFU of ST2 from the mixed cultures, measured by subtracting the ST59 CFU from the total grown on the untreated TSA plates. Each bar represents the mean and standard deviation of three or four replicates. *t* tests were used to compare mean CFU of each strain in the pure and mixed cultures (*, *P* < 0.05). Download FIG S2, PDF file, 0.1 MB. Copyright © 2021 Hellmann et al. 2021 Hellmann et al. https://creativecommons.org/licenses/by/4.0/ This content is distributed under the terms of the Creative Commons Attribution 4.0 International license.

### An episode of suspected infection and rapid growth of an S. epidermidis strain.

During weeks 5 through 8, one of the infants had belly distention, increased oxygen needs due to desaturation, as well as increased endotracheal tube secretions, mildly elevated C-reactive protein, and decreased urine output. Abdominal X-ray was suggestive of pneumatosis intestinalis, and hence the infant had suspected necrotizing enterocolitis. Blood cultures were negative, but Stenotrophomonas maltophilia bloomed to 92% relative abundance in the infant’s week 6 stool sample and was also detected at <1% relative abundance in the infant's week 6 nasal swab. This infant did not produce stool during week 8 sampling, but the week 8 nasal swab again yielded S. maltophilia at 24% relative abundance. S. maltophilia occurred in only one other sample in our study, in a different infant and time point at <1% relative abundance in stool. The infected infant was treated with vancomycin-amikacin and with ceftazidime as outlined in Fig. 3 (bottom panel, infant 1). The metagenomic assembly of S. maltophilia showed that this strain was related to multilocus sequence type 140 and that it carried resistance determinants to all antibiotic classes used for treatment as follows: Gram-negative cell wall (intrinsic glycopeptide resistance), *aph*(3′)-IIc (aminoglycoside resistance), *blaL1* (beta-lactam resistance), and efflux pumps active against six classes of antibiotics.

**FIG 7:**
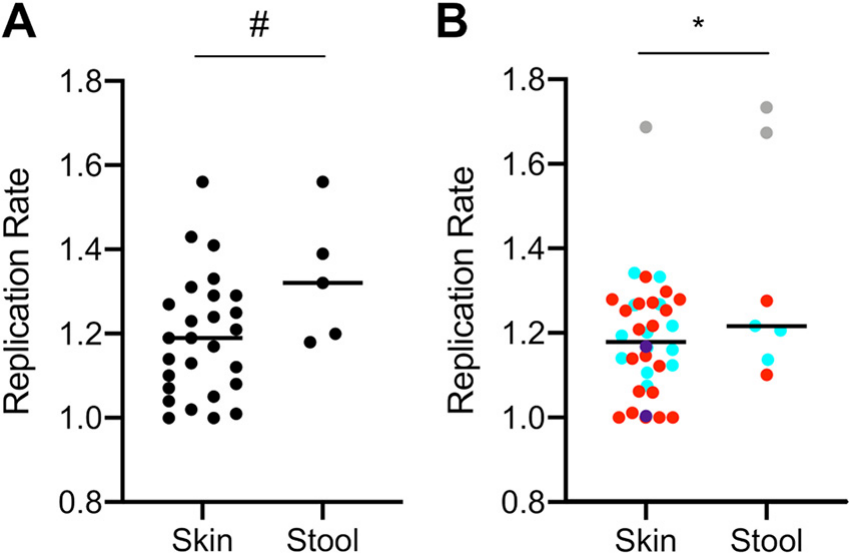
Replication rates of S. epidermidis from preterm infant skin and stool samples. (A) Species-level replication rates of S. epidermidis as measured by GRiD. (B) Strain-level replication rates of S. epidermidis as measured by SMEG. ST2 is represented in red, ST59 is represented in cyan, ST5 is represented in gray, and purple represents two rare STs that appeared in only a single sample. *t* tests were used to compare mean replication rate between body sites (#, 0.1 > *P* > 0.05; *, *P* < 0.05).

The rare ST5 strain of S. epidermidis was also detected in this infant’s week 6 stool sample, and it achieved the highest replication rate among all S. epidermidis samples based on GRiD (1.56; mid-exponential phase in Fig. 1B and C) and SMEG (1.73; off-scale in Fig. 1B and C). The replication rate of S. epidermidis in stool samples tended to be higher than in skin swabs (*t* test for GRiD values, *P* = 0.056) (Fig. 7A). This growth difference by body site was also reflected at the strain-level (*t* test for SMEG values, *P* = 0.038) (Fig. 7B). However, the highest replication rates at both body sites were attributed to ST5 (Fig. 7B, gray), and the significant difference in mean replication rate between body sites disappeared when the ST5 values were removed (*t* test for SMEG values, *P* = 0.74). Since blood cultures were negative during this episode, it was not clear whether the S. maltophilia or S. epidermidis strains, or some other pathogen, was responsible for the infant’s clinical symptoms. However, our data placed an unusually rapidly growing S. epidermidis strain in stool in temporal association with this episode.

## DISCUSSION

At >6 weeks postbirth in healthy children, the microbiome of different body sites becomes differentiated and personalized (36, 37). In healthy adults, there are differences in the distribution of CoNS species, and even strains of S. epidermidis, across skin sites (17, 22, 38). However, different skin sites of preterm infants, and especially extremely low-birthweight infants, may not be physiologically differentiated by 6 weeks postbirth and may remain more moist than adult skin (18, 39–41). Skin microbiome development is therefore delayed in preterm infants, and it is likely to be influenced by birth mode, feeding habit, antibiotic exposure, and the NICU environment (36, 37, 40, 42–44).

In this study, the preterm infant skin microbiome was dominated by S. epidermidis and *S. capitis*, both of which are major causes of antimicrobial-resistant infections of this vulnerable host population (3, 4, 6, 7). We did not observe statistically significant differences in the microbiome diversity or composition of different skin sites. Our use of a marker-based species identification method (MetaPhlAn2) has high specificity but low sensitivity relative to some other methods (45). Thus, while we can be confident in the identification of most species, we have likely missed some species that would be present at low frequencies. Nonetheless, we did observe the development of more diverse microbial communities with different species on the skin of these infants over time even though each infant was treated with multiple courses of antibiotics during this study. Despite extensive sharing of species across body sites, the microbiome composition of skin and stool was distinguished from each other, signaling the beginning of microbiome differentiation by body site.

Multiple methods of strain identification indicated that populations of the most prevalent species, S. epidermidis, were composed of two predominant strains. Our results provided evidence for antagonism between S. epidermidis strains ST2 and ST59. On skin, these strains cooccurred with different species and replicated fastest in different samples. ST2 colonized skin earlier but was often replaced by ST59 over time. Under laboratory conditions, we found that reference isolates of ST2 produced more biofilm than ST59, and biofilm was reduced during mixed culture. Although the laboratory experiments showed the feasibility of a direct antagonism between ST2 and ST59, there are limitations to this interpretation in the preterm infant context because the strains used were not isolated from the infants and potential indirect factors, such as microenvironment modifications, were not considered. Prior work has shown that these strains are more common from hospitalized persons than from nonhospitalized persons (12–14). ST2 is a well-known hospital-specialist strain that causes antibiotic-resistant infections worldwide (46–48). ST59 is less studied but is also known to be antibiotic resistant (49).

Metagenomic assembly showed that the ST2 and ST59 strains encoded identical *agr* type I sequences at the amino acid level. The *agr* quorum-sensing system is a global regulator of virulence gene expression, and amino acid polymorphisms distinguish at least three *agr* types in S. epidermidis that are thought to inhibit each other’s expression (50). In healthy adults, cocolonization of skin with S. epidermidis strains of different *agr* types might be common, which should lead to less virulent populations (51). Conversely, cocolonizing strains of the same *agr* type, as occurred in our study, would be expected to result in increased activation of *agr* with associated increased expression of degradative enzymes and toxins and decreased expression of biofilm (52). Regardless of whether the mechanism of antagonism is direct or indirect and whether *agr* has a role, our study shows that antagonism between S. epidermidis strains can occur even when they are of the same *agr* type.

PMA treatment of microbiome samples to enrich for the viable microbiome was a novel aspect of this study. While not guaranteed to be equally effective for removing nonviable DNA of all microbes, we showed that PMA treatment of laboratory cultures effectively removed DNA from nonviable S. epidermidis. The ∼5 × 10^8^ CFU/ml of S. epidermidis used in these experiments exceeds real world microbial biomass on infant skin by many orders of magnitude. For example, in one study, the baseline number of Gram-positive bacteria from 2 cm^2^ swabbing (as done in our study) of the arm and groin of neonates averaged 155 and 65 CFU/ml, respectively (53). Furthermore, when examining PMA’s ability to remove purified genomic DNA of S. epidermidis, we used ∼250 ng in experiments but the skin samples that we processed had an average of <8 ng of total DNA.

Another approach to focus study on the viable microbiome is to consider microbial growth *in situ*. Because bacterial chromosomes replicate from an origin to a terminus and multiple rounds of replication can be initiated before prior rounds are completed, the copy number of sequences near the origin compared to sequences near the terminus gives information about replication rate. Previous work using this approach showed active replication of S. aureus in nasals swabs of adult volunteers (54). Here, we showed that PMA treatment of swabs did not interfere with these estimates and was even required to obtain an accurate estimate of replication rate for S. epidermidis. Another study of bacterial replication rates within the preterm infant microbiome found that species from (a few) skin samples grew faster than the same species from stool samples (18). Our results based on viability treatment show the opposite result, with a faster replication rate for S. epidermidis in stool versus that in skin. However, this result was attributed to the faster growing ST5 strain. The ability of S. epidermidis strains to reach high relative abundance in stool has been pointed out previously (16), but the clinical relevance of this ability needs to be established.

One limitation of this study is that we measured relative abundance of microbes rather than their absolute abundance. Density-dependent phenomena, such as bacterial growth and quorum-sensing, that impact species interactions might be best evaluated with absolute abundance data. For example, Liu et al. (55) showed that absolute abundance data was critical for detecting interactions of S. aureus with other species of the nasal microbiome of adult twins. More recently, interactions of S. epidermidis with K. pneumoniae in preterm infant stool were detected with use of absolute abundance data (20). Another limitation of this study was the 2-week time period between microbiome sampling. Colonization dynamics occurring more frequently than 2 weeks could be missed with this sampling scheme.

The level of sequencing done in this study (on average, 5.9 M reads for skin swabs and 22.6 M reads for stool samples) falls in between shallow sequencing (0.5 M reads) and deep sequencing (50 M reads) following the scheme of Hillmann et al. (56). This depth of coverage was adequate to reliably identify two predominant strains of S. epidermidis but was less successful with metagenomic assembly of these strains. Besides depth of coverage, breadth of coverage is an important consideration for complex microbial communities (57). The preterm infant skin microbiome appears to be much less complex than that of healthy infants or adults, with the predominance of a few species that can thrive in an NICU setting (18, 44, 58). Thus, moderate amounts of shotgun sequencing data appear to be adequate for characterizing interactions among predominant strains in this patient population.

## MATERIALS AND METHODS

### Infant enrollment and sample collection.

This study was approved by the institutional review board of the University of Mississippi Medical Center. During spring and summer 2019, four extremely low-birthweight infants were enrolled within their first week of life following maternal consent. Infants with major congenital malformations, including craniofacial abnormalities and known or suspected chromosomal anomalies or syndrome, were excluded. Five skin sites (axillary vault, inguinal crease, nares, periumbilical region, and upper chest) were swabbed using nylon, flocked, minitip swabs (Copan Diagnostics) premoistened with sterile phosphate-buffered saline (PBS), in 2- by 2-cm patches, with 10× swabbing motion. Nares swabs were circled 10×. If stool from a diaper was available within the day of swabbing, 0.5 mg of the stool was collected. The samples were collected biweekly for the infants’ first 2 months of life. Swab heads, removed with sterile scissors, and stool were suspended in 500 μl sterile, diethyl pyrocarbonate (DEPC)-treated water, vortexed briefly, incubated at room temperature for 5 min, and vortexed again (21). The samples were immediately processed according to the PMA treatment protocol outlined below and then were frozen at −20°C for subsequent DNA extraction.

### PMA treatment of samples.

In the dark, PMA (Biotium) was added to samples to a final concentration of 50 μM. Samples were briefly vortexed and then shaken at room temperature for 5 min prior to light exposure. Samples were laid horizontally on a layer of aluminum foil over ice, 20 cm underneath a benchtop fluorescent light of 32 W, and incubated for 25 min under the light, vortexing briefly every 5 min.

### Testing the effects of PMA treatment.

S. epidermidis ST2 strain DAR1907 (59) and S. aureus ST8-USA300 strain JE2 (60) were plated for isolation on tryptic soy agar (TSA) plates. A single colony was inoculated into 5 ml of tryptic soy broth (TSB) and grown at 37°C to an optical density at 600 nm (OD_600_) of 0.6. S. epidermidis was heat-killed at 85°C for 1 h, and 100 μl was plated to check for viability. S. aureus was divided into eight 125-μl and 250-μl aliquots. A total of 125 μl of the heat-killed S. epidermidis was added to the 125 μl of the live S. aureus aliquots. Purified S. epidermidis genomic DNA was added to the 250 μl of the live S. aureus to a final concentration of 1 ng/μl S. epidermidis genomic DNA. Half of the samples were then treated with PMA before freezing at −80°C.

S. epidermidis DAR1907 was plated for isolation on TSA plates, and a single colony was inoculated into 5 ml of TSB. This culture was grown overnight to saturation, and 500 μl was used to inoculate 20 ml of fresh TSB and vortexed. The 250-μl aliquots were added to wells of a flat-bottom, polystyrene, 96-well plate (Costar). The plate was incubated at 37°C with slow shaking on a Synergy 4 microplate reader (BioTek), taking OD_600_ measurements and removing 8 samples every hour. Half of the samples were treated with PMA and the other half left untreated before freezing at −80°C.

### DNA extraction, concentration, and sequencing.

DNA was extracted from the infant microbiome samples using the MagAttract PowerSoil DNA isolation kit (Qiagen) for the KingFisher flex instrument (Thermo Fisher), according to the manufacturer’s protocol. Subsequently, 0.1× 3 M sodium acetate and 2.5× chilled 100% ethanol, plus RNA-grade oyster glycogen (Thermo Fisher) at a final concentration of 0.5 μg/μl, were added to the samples. Samples were vortexed briefly before incubating at −20°C for 1 h. The samples were centrifuged at 12,000 rpm for 15 min, and the supernatant was discarded. The samples were washed with 200 μl of chilled 70% ethanol before being centrifuged and the supernatant discarded again. The samples were allowed to dry completely before being resuspended in 12 μl of DEPC-treated water. Double-stranded DNA (dsDNA) was quantified using the Qubit high-sensitivity assay (Invitrogen).

Experimental samples had DNA extracted with the DNeasy blood and tissue kit (Qiagen). Fifteen units of lysostaphin and 1,500 units of lysozyme were added with 1× TE buffer up to 200 μl to the samples and incubated at 37°C for 15 min. Twenty microliters of proteinase K and 200 μl of AL buffer were added and the samples incubated at 68°C for 15 min. The samples were added to the spin columns and washed and eluted following the manufacturer’s protocol.

Sequencing libraries were prepared using Illumina’s Nextera XT protocol automated with a Zephyr G3 instrument (Perkin Elmer). Samples with libraries of <0.09 ng/μl were not sequenced. PhiX control was added to libraries at 1% prior to sequencing. Infant samples were sequenced using Illumina’s 300 cycle NextSeq 500/550 high-output kit v2.5. Negative controls that were treated exactly as the infant samples were included in each run of the NextSeq. Experimental samples were sequenced using Illumina’s 500 cycle MiSeq reagent kit v2.

### Metagenomics analyses.

**(i) Species identification.** Sequence reads were processed with KneadData v0.7.3 from the bioBakery analysis pipeline (61) to remove human, PhiX, and 16S rRNA reads. Trimmomatic (62) options were modified to SLIDINGWINDOW:4:12 and MINLEN:30. MetaPhlAn2 v2.9.5 (28) was used to identify species, relative abundance, and estimated number of reads per species using the CHOCOPhlAn database v925.

**(ii) Strain identification.** MIDAS v1.3.2 (33) was used with its database v1.2 to identify strains in samples with >3.5× S. epidermidis coverage (>82,000 reads) as estimated by MetaPhlAn2, as this method seemed to have a higher coverage requirement. StrainEst v1.2.4 (16) and SMEG v1.1 (30) were used to identify strains on samples with >1× S. epidermidis coverage (>24,000 reads). For StrainEst, only strains with >1% relative abundance were considered. The SMEG default database for S. epidermidis was used with T.0.5 and the *de novo* methodology.

**(iii) Replication rate estimation.** Species-level replication rates were estimated using GRiD v1.3 (29) on samples with >1× S. epidermidis coverage (>24,000 reads) as estimated by MetaPhlAn2. The complete genome of S. epidermidis DAR1907 was used as the reference for read mapping, and samples with a heterogeneity score of >0.3 were removed. GRiD is effective for coverages as low as 0.2× (29); however, we restricted analysis to samples with >1× coverage because heterogeneity scores increased, and inconsistencies in results using different reference genomes were noticed at lower coverages. SMEG was used to obtain strain-level replication rate estimates and coverages with the settings described above.

**(iv) Diversity and ecological interactions.** Alpha and beta diversity and species and S. epidermidis strain correlations were studied using MicrobiomeAnalyst (63). Species occurring in only a single sample were removed for diversity analyses. Alpha diversity was calculated using the Simpson index, and beta diversity was calculated using the Jaccard index. PERMDISP was used to test the homogeneity of group variances, and PERMANOVA was used to test the differences between mean beta diversity of groups. SparCC (34) was used to correlate the relative abundance of species and S. epidermidis strains as identified by StrainEst. Relative abundance from Metaphlan2 was normalized to 100,000 reads and species occurring in <10% of samples were removed.

**(v) Metagenomic assembly and gene identification.** Reads of each sample were assembled *de novo* into contigs with SPAdes v3.11.1 (64, 65) and mapped onto the contigs with bwa v0.7.12. Contigs were binned with MetaBAT2 v2.15 (66). Bin quality was evaluated with CheckM (67), and genomes with >70% completeness and <5% contamination were retained (68). MLST (69, 70) was used to identify sequence types. BLASTN was used to identify S. epidermidis *agr* types and the presence of the *ica* operon. ABRicate v1.0.1 (71) was used to identify resistance genes with the CARD and NCBI databases (72, 73).

### Validating strain interactions.

Biofilm assay followed the procedures of O’Neill et al. (74). Briefly, S. epidermidis ST2 strains DAR1946 from New York (59) and DAR4887 from Mississippi (unpublished) and S. epidermidis ST59 strain DAR2087 from New York (59) were grown for single colonies on TSA and then inoculated into 5 ml of brain heart infusion (BHI) broth. The bacteria were grown 24 h at 37°C before adjusting the broth to an OD_600_ of 1. Fifty microliters of each strain were added to BHI up to 1,000 μl either alone or in a mixture of ST2 and ST59. The samples were vortexed and plated in eight replicates on Nunclon delta-treated, flat-bottom, polystyrene, 96-well plates (Thermo Fisher). Plates were incubated at 37°C for 24 h before discarding the broth and gently washing three times with 100 μl sterile water. Plates were dried at 61°C for 1 h and stained with 100 μl 0.4% crystal violet for 10 min. Plates were washed three times with 200 μl sterile water and the absorbance measured at 492 nm.

CFU within the biofilm were quantified by scraping the 24-h biofilm and suspending in 100 μl sterile water. The biofilm scrapings were sonicated with a Aquasonic 75T ultrasonic cleaner (VWR Scientific) for 5 min and vortexed on high for 30 s. Four serial dilutions were plated on TSA plates and TSA plates containing 10 μg/μl erythromycin (Sigma) and incubated at 37°C for 48 h. The ST59 strain was erythromycin resistant and the two ST2 strains were erythromycin susceptible.

### Statistical analysis.

Prism v8.3.0 (GraphPad) was used for most statistical analysis. Differences between group means were tested by *t* test (2 groups) or ANOVA (>2 groups simultaneously) after testing for homogeneity of variances and log transformation if necessary to give similar variances. Correlations used Pearson’s coefficient. Statistical significance was achieved at a *P* value of <0.05, and trends were noted at 0.1 > *P* > 0.05.

### Data availability.

The KneadData-filtered sequence reads are deposited in the NCBI SRA database under BioProject accession number PRJNA748448. The relative abundance of species has been made available as Table S2 in the supplemental material.
