# Supplementary figures and images for: Viability-Resolved Metagenomics Reveals Antagonistic Colonization Dynamics of Staphylococcus epidermidis Strains on Preterm Infant Skin
Source: mSphere. 2021 Sep 15;6(5):e00538-21. doi: 10.1128/mSphere.00538-21 (PMC8550141; doi:10.1128/mSphere.00538-21)

A

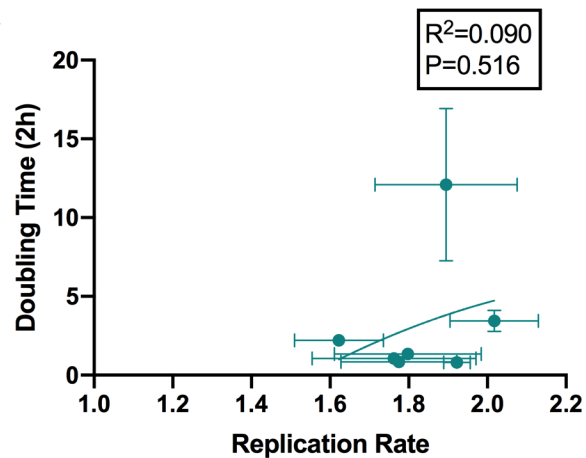

B

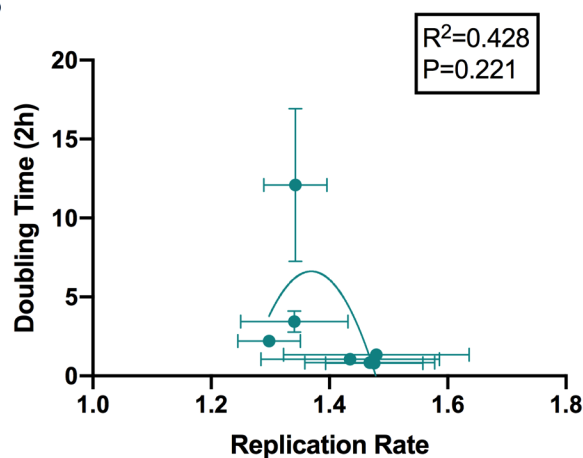

C

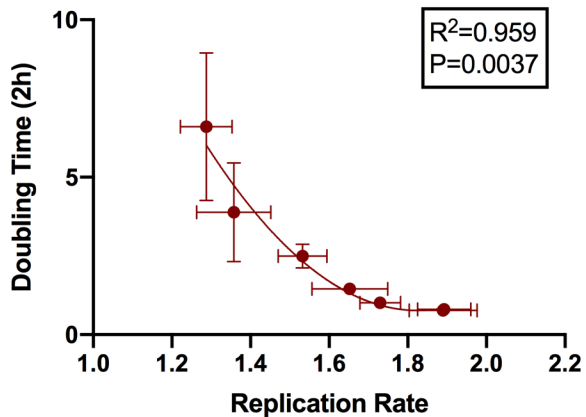

D

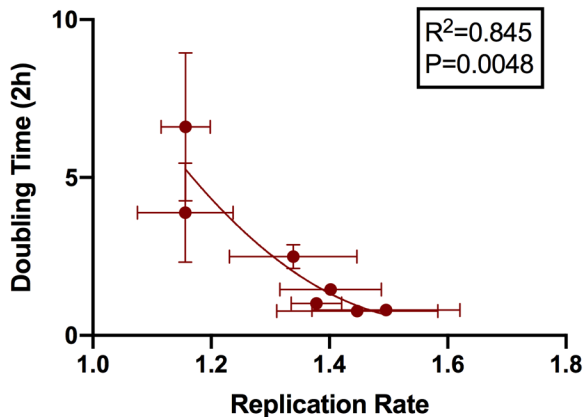

Supplement: FIG S1 [file msphere.00538-21-sf001.pdf]

A

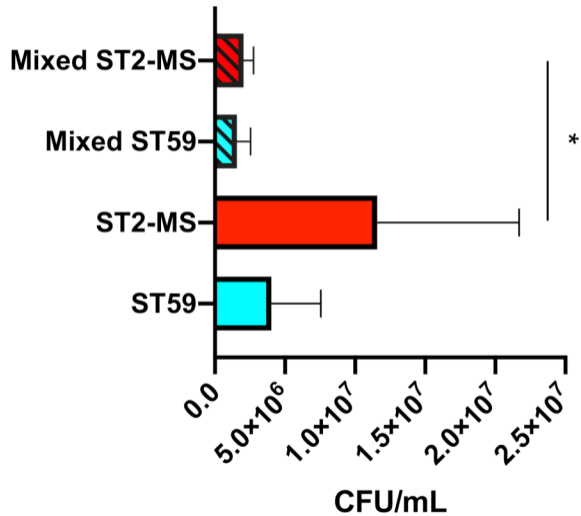

B

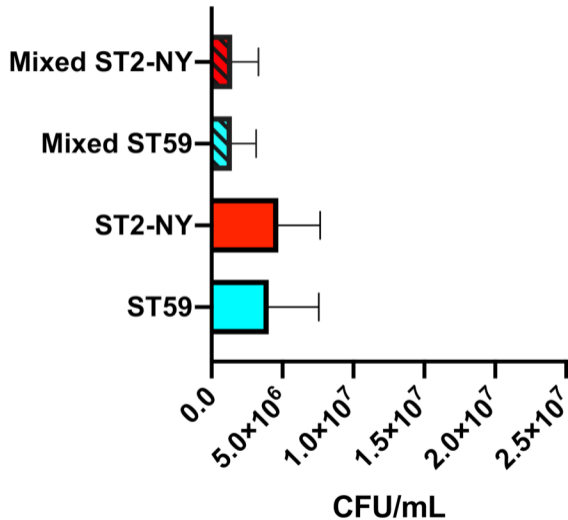

Supplement: FIG S2 [file msphere.00538-21-sf002.pdf]
